# Protocol for the development and validation of a Core Set for exercise-based rehabilitation of adults with multiple long-term conditions (multimorbidity) based on the World Health Organization’s International Classification of Functioning, Disability, and Health (ICF) framework

**DOI:** 10.1177/26335565251343923

**Published:** 2025-05-20

**Authors:** Fanuel M. Bickton, James R. Manifield, Felix Limbani, Justin Dixon, Anne E. Holland, Rod S. Taylor, Claire Calderwood, Walter Wittich, Celia L. Gregson, Martin Heine, Zahira Ahmed, Ronel Roos, Sally J. Singh, the Africa Multimorbidity Alliance – Multimorbidity Rehabilitation Working Group

**Affiliations:** 1 Lung Health Research Group, 560808 Malawi-Liverpool-Wellcome Programme, Blantyre, Malawi; 2 Department of Rehabilitation Sciences, School of Life Sciences and Allied Health Professions, Kamuzu University of Health Sciences, Blantyre, Malawi; 3 Centre for Exercise and Rehabilitation Science, Leicester Biomedical Research Centre (BRC)– Respiratory, University Hospitals of Leicester NHS Trust, National Institute for Health and Care Research (NIHR), Leicester, UK; 4 Department of Respiratory Sciences, 574216 University of Leicester, Leicester, UK; 5 The Health Research Unit Zimbabwe, 559150 Biomedical Research and Training Institute, Harare, Zimbabwe; 6 Department of Global Health & Development, 4906 London School of Hygiene and Tropical Medicine, London, UK; 7 Department of Respiratory and Sleep Medicine, Alfred Health, Melbourne, VIC, Australia; 8 School of Translational Medicine, 2541 Monash University, Melbourne, VIC, Australia; 9 Institute for Breathing and Sleep, Heidelberg, VIC, Australia; 10 MRC/CSO Social & Public Health Sciences Unit, Institute of Health and Wellbeing, 3526 University of Glasgow, Glasgow, UK; 11 Robertson Centre for Biostatistics, Institute of Health and Wellbeing, 3526 University of Glasgow, Glasgow, UK; 12 Clinical Research Department, 4906 London School of Hygiene and Tropical Medicine, London, UK; 13 School of Optometry, 5622 Université de Montréal, Montreal, QC, Canada; 14 Global Health and Ageing Research Unit, Bristol Medical School, University of Bristol, Bristol, UK; 15 Department of Global Public Health and Bioethics, Julius Center for Health Sciences and Primary Care, University Medical Center Utrecht, 8125 Utrecht University, Utrecht, The Netherlands; 16 Institute of Sport and Exercise Medicine, Department of Exercise, Sport and Lifestyle Medicine, Faculty of Medicine and Health Sciences, Stellenbosch University, Cape Town, South Africa; 17 Department of Physiotherapy, School of Therapeutic Sciences, Faculty of Health Sciences, 37707 University of the Witwatersrand, Johannesburg, South Africa

**Keywords:** core outcome sets, ICF core sets, functioning, multimorbidity, rehabilitation

## Abstract

**Background:**

Core outcome sets for people with multiple long-term conditions (multimorbidity) intervention studies offer an opportunity to compare data across studies and countries. However, a key research gap remains: the development of the World Health Organization (WHO) International Classification of Functioning, Disability and Health (ICF) Core Set for multimorbidity rehabilitation. ICF Core Sets are a selection of essential categories from the full ICF classification that are considered most relevant for describing the functioning of a person with a specific health condition or in a specific healthcare context. This study aims to develop and validate an ICF Core Set for exercise-based multimorbidity rehabilitation. Unlike system- or disease-specific rehabilitation, multimorbidity rehabilitation entails using a modified structure that accommodates all conditions that an individual with multimorbidity has.

**Methods:**

The three-phase, multi-method process created by the WHO and ICF Research Branch will be followed. The process will involve conducting four preparatory studies (phase 1), including (i) a systematic literature review (to examine researcher perspectives), (ii) a qualitative study (to examine patient perspectives), (iii) an expert survey (to examine health professional perspectives), and (iv) an empirical study (to examine clinical perspectives). This will be followed by an international consensus conference (phase 2) where lists of ICF categories resulting from phase 1 studies will be consolidated into a first version of an ICF Core Set for multimorbidity rehabilitation, which will be validated using an international comparative data analysis (phase 3).

**Conclusion:**

An ICF Core Set created for multimorbidity rehabilitation will (1) benefit patients with multimorbidity who are often excluded from clinical trials of single-disease rehabilitation programs, (2) ensure precise and comprehensive assessment and documentation of functioning and disability relevant to this patient population, (3) help rehabilitation providers and their patients and/or caregivers when setting rehabilitation goals and planning rehabilitative interventions to achieve those goals, (4) help researchers in the synthesis of evidence for multimorbidity rehabilitation and facilitate the comparability of data across studies and countries, and (5) provide the scientific basis from which assessment tools can be derived for use in clinical and research settings and health care administration.

**Trial Registration:**

COMET database (https://www.comet-initiative.org/Studies/Details/3266).

## Background

Chronic conditions are health problems that require ongoing management over a period of years or decades and can be physical or mental, communicable or non-communicable.^1^ These conditions often co-exist in one individual, termed ‘multiple long-term conditions’ or ‘multimorbidity’.^2^ Evidence from high-income countries (HICs) has shown that social deprivation is associated with higher multimorbidity prevalence.^3^ About a fifth of adults in low- and middle-income countries (LMICs) are living with multimorbidity, and prevalence may already surpass that seen in HICs.^4^ In addition to the rising prevalence of non-communicable diseases, multimorbidity in LMICs is increasing and includes the high burden of chronic infectious diseases such as chronic human immunodeficiency virus (HIV) infection.^5^

Compared to people living with single chronic conditions, people with multimorbidity are at risk of increased healthcare utilization, extended hospital admission, poorer physical and psychosocial health, polypharmacy, and premature death.^6^ Multimorbidity often leads to decline in physical function and is associated with disability.^1,7^ Interventions designed to target difficulties that people with multimorbidity experience may, therefore, be effective.^1^ This includes rehabilitation – that is, a set of interventions needed when a person is experiencing limitations in everyday physical, mental, and social functioning due to ageing or a health condition, including chronic diseases or disorders, injuries, or trauma.^8^ Chronic disease rehabilitation includes exercise-based rehabilitation.^2^ A recent overview of systematic reviews found evidence that participation in exercise-based rehabilitation was beneficial in 25 out of the 45 pre-specified chronic conditions, supported by improvements in health-related quality of life (HRQoL) and exercise capacity.^9^

The World Health Organization (WHO) acknowledges that access to rehabilitation is a human right,^10^ as enshrined in the United Nations Convention on the Rights of Persons with Disabilities.^11^ Unfortunately, significant health system challenges exist in rehabilitation service provision, particularly in low-resource settings (including shortage of rehabilitation professionals and assistive devices)^12^ where contextual factors including social inequities and childhood adversity increase the risk for multimorbidity.^13^ Additionally, when available, rehabilitation is frequently siloed within single-disease programs such as cardiac or pulmonary rehabilitation.^14,15^ In reality, many patients attending such single-disease rehabilitation programs have multiple, complex health conditions.^16^ Single-disease care may not be appropriate or accessible for people with multimorbidity, due to the potential interaction between diseases and drugs as well as total treatment burden.^16^ This necessitates the need for rehabilitation professionals to shift toward a ‘multimorbidity rehabilitation’ model which, unlike system- or disease-specific rehabilitation models, entails using a modified rehabilitation structure that accommodates all conditions that a patient with multimorbidity has, and thus does not limit participation in rehabilitation to a specific disease.^17–19^

A growing body of research is examining the effectiveness of interventions, including exercise-based rehabilitation, to address the specific experiences of patients with multimorbidity.^1,2,20–23^ However, due to heterogeneity in outcome measurements between these studies, evidence synthesis by systematic reviews is challenging.^24^ One solution is for studies to use a core outcome set (COS). A COS is an agreed minimum set of outcomes to be measured in all trials of a specific intervention for health condition(s).^25,26^ A COS can also include specific measures of each outcome^27^ and can facilitate outcome consistency across studies and consistent reporting in clinical research.^28^ Evidence synthesis on the effectiveness of various health interventions for people with multimorbidity could benefit from a wide and structured implementation of COS in clinical trials.^28^

Both globally oriented and LMIC-specific COS for multimorbidity intervention studies have been developed.^29,30^ Smith et al. used a Delphi consensus process to develop their COS for multimorbidity research (COSmm).^29^ A set of 17 outcomes were agreed upon, with quality of life, mental health outcomes, and mortality prioritised highest. Notably, some of these outcomes were adopted in the eligibility criteria for study selection in a systematic review and meta-analysis of randomised controlled trials investigating the benefit of exercise-based rehabilitation in people with multimorbidity.^2^ However, the COSmm study had several limitations including being based on expert input primarily from HICs and not directly involving patient representatives in developing the initial COSmm. Intending to address these limitations, Vidyasagaran et al. developed a COS for trials of interventions for multimorbidity prevention and treatment, specific to adults in LMICs (COSMOS).^30^ The COSMOS also included qualitative interviews with people with multimorbidity and their caregivers living in LMICs to generate the initial COS.

Noteworthy, both COSmm and COSMOS included broad concepts of (HR)QoL. While important to include, there is considerable heterogeneity in the way (HR)QoL is defined and operationalised, which limits comparability and interpretation of (HR)QoL scores and complicates literature searches.^31^ An attempt by both studies to more precisely define (HR)QoL would likely have been hindered by the multiple ways that (HR)QoL is conceptualised in the domains and the items included in the various instruments they identified. This was evident in COSmm study where, due to the wide range of potential outcome metrics included in their Delphi survey (round 1), participants found it difficult to agree on specific outcome metrics (e.g., HRQoL Short-Form-36 or EuroQol/EQ-5D questionnaire); hence, only outcome domains without specific outcomes measures were proposed.

Rehabilitation professionals (including those among our author group^20^) would find it difficult to operationalise many of the broad concepts included in the existing COS for multimorbidity, particularly (HR)QoL. These professionals must often set SMART (specific, measurable, attainable, realistic and time-bound) rehabilitation goals with their patients and/or caregivers based on the WHO’s International Classification of Functioning, Disability and Health (ICF) framework. In addition, we do not know how adequate and relevant the current COSs are to functioning, which is a key indicator for rehabilitation’s performance and impact on a person’s health state and lived experience of health.^32^

**Figure 1.**
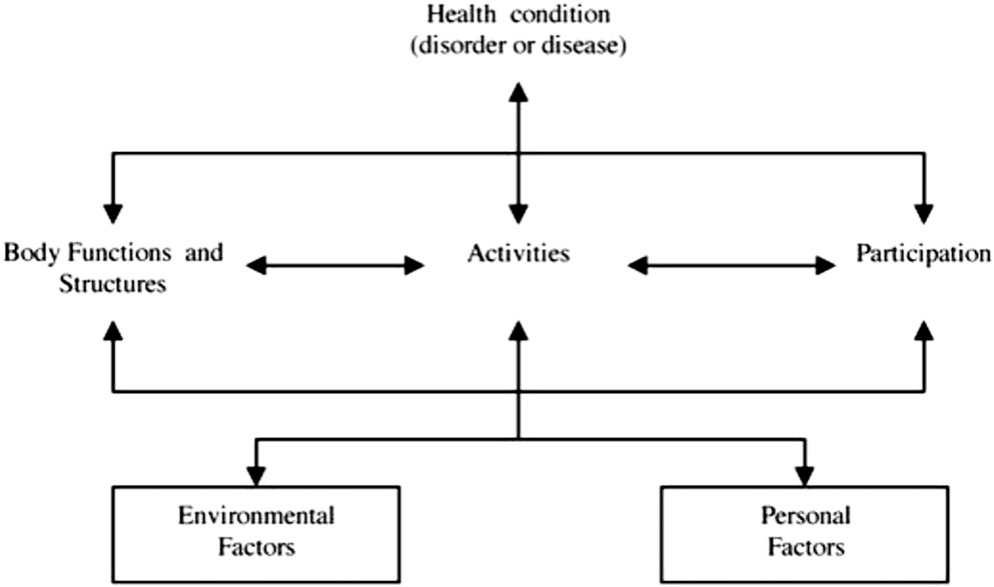
Interactions between components of the ICF^36^.

These gaps can be addressed by developing ‘ICF Core Sets’ for multimorbidity rehabilitation. Unlike COS, ICF Core Sets are a selection of essential categories from the full ICF classification that are considered most relevant for describing the functioning of a person with a specific health condition or in a specific healthcare context.^33^ In other words, ICF Core Sets can serve as a minimal standard for the assessment and reporting of functioning and health in clinical practice and studies.^33^ The WHO argues that the concept of functioning (body functions and structures, activities, and participation) best captures our intuitive notion of health.^34^ Optimal functioning is, therefore, a major public health goal.^34^ The ICF presents a unified and consistent language of human functioning suitable as a reference for comparing health information. Functioning, as defined in the ICF, includes the physiological and psychological functions of body systems, plus the tasks and actions of individuals and their involvement in all life situations.^34^ Figure 1 depicts the components of the ICF and their interactions. The health condition may impact functioning at three mutually interacting levels: in relation to the body, at the level of activities, and at the level of participation in society.^35^ How a health condition impacts functioning should also be considered within the context of environmental and personal factors.^35^

To facilitate the rehabilitation process, ICF Core Sets have been developed for over thirty health conditions including neurological (e.g., cerebral palsy), respiratory (e.g., chronic obstructive pulmonary disease), cardiovascular (e.g., stroke), mental health (e.g., depression), musculoskeletal (e.g., low back pain), and other conditions (e.g., amputation, hearing loss) (https://www.icf-research-branch.org/). These ICF Core Sets focus on single conditions’ impact on functioning. However, most people with a chronic condition have multimorbidity, and a single-condition-aimed ICF Core Set may miss relevant difficulties in functioning that arise from the interaction of conditions in the context of multimorbidity.^37^

### Protocol aim

This protocol describes the three-phase, multi-method process for developing and validating an ICF Core Set for rehabilitation of adults with multimorbidity.

## Methods

**Figure 2.**
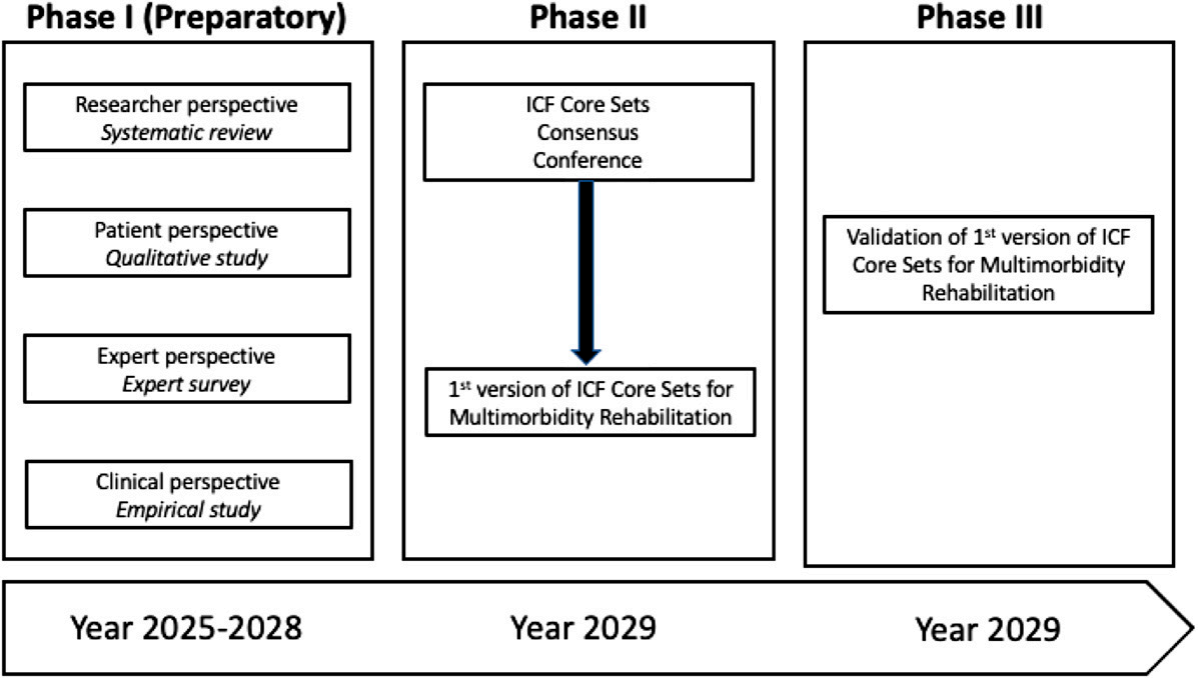
Time schedule of the process to develop and validate ICF Core Sets for multimorbidity rehabilitation.

We will follow a three-phase, multi-method process created by the WHO and ICF Research Branch^33^ to develop the first version of the ICF Core Set for multimorbidity rehabilitation (Figure 2). The process will involve conducting four preparatory studies (phase 1), including (i) a systematic literature review (to examine researcher perspectives), (ii) a qualitative study (to examine patient perspectives), (iii) an expert survey (to examine the health professional perspectives), and (iv) an empirical study (to examine clinical perspectives). These studies will provide the evidence base for an international consensus process to establish the first version of the ICF Core Set (phase 2). This version will be validated in phase 3 in an international comparative data analysis.

The conduct of this research project will conform to the guidelines of the World Medical Association (WMA) Declaration of Helsinki – ethical principles for medical research involving human participants. Ethical approvals for the qualitative study (patient perspective) and the empirical multicentre study (clinical perspective) will be sought from the local ethics review boards in participating countries (institutional review boards at the affiliations of the respective co-investigators). Informed consent will be obtained from all participants in the study; written or thumbprint or witnessed verbal consent will be acquired for those participants in the qualitative interviews. Local co-investigators/collaborators will facilitate these ethical clearances. In the qualitative study, inclusion of caregivers (or family members) of people with multimorbidity will help prevent exclusion of individuals with insufficient decisional capacity such as those with cognitive impairments. This is because caregivers can provide researchers with proxy consent and data about such individuals.^38^ For online expert survey rounds, study information, and privacy and terms of agreement for participating, will be given to participants at the start. This will be followed by a statement “by continuing, you are giving informed consent to participate.” For the validation phase, data sharing agreements will be sought from the data custodian institutions (through principal investigators) of the Multilink, COSMOS, and PERFOM studies. The globally influential standards of the European Union’s General Data Protection Regulation (GDPR), and similar provisions of non-European participants’ countries, will be followed to ensure participants’ data protection/privacy.

There may be subsequent amendments to and deviations from this original protocol after the study commences, and it is difficult to foresee them. We will be guided by item 10 of the CONSORT (Consolidated Standards of Reporting Trials) statement^39^ in handling them if they occur. Any substantial amendments to the protocol will be submitted to the Steering Committee and ethics review boards for their review and approval. A possible protocol deviation would be to conduct phase 2 (international consensus conference) physically if resources allow (e.g., with acquisition of further funding), instead of online as currently proposed. Another possible protocol deviation would be to perform an LMIC-focused systematic review for preparatory study 1 (researcher perspective) to supplement the currently proposed secondary analysis of the overview of systematic reviews, if data in those systematic reviews underrepresent LMICs. Any protocol deviation will be fully and clearly documented in the final report, along with the rationale for those changes, to help the reader interpret the results. Due to the non-interventional design of this study, no safety risks associated with protocol deviations are anticipated.

### Phase 1

#### Preparatory study 1: Systematic literature review (researcher perspective)

**Figure 3.**
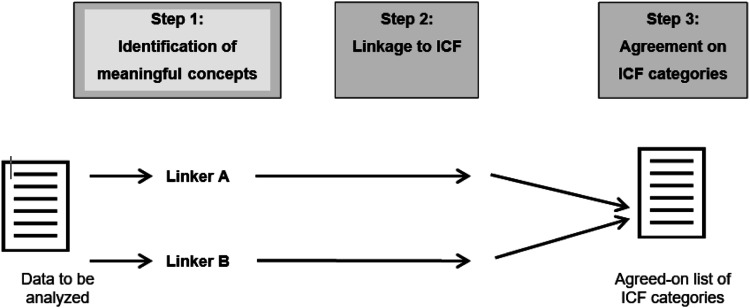
General overview of the ICF linking process (adapted from a template for study protocols regarding ICF Core Set development by the ICF Research Branch).

In a prior secondary analysis of the overview of systematic reviews,^9^ JM extracted outcome measures used within exercise-based rehabilitation interventions for people with multimorbidity, from studies included in two recent systematic reviews by Bricca et al.^2^ and Barker et al.^23^ In this phase, we will use ICF to identify and quantify meaningful concepts contained in those outcome measures through an established ICF linking process.^40^ Meaningful concepts are concise descriptions of specific behaviours, skills or other aspects of functioning that are to be linked to ICF categories.^41^ Two reviewers will independently link each meaningful concept to the ICF categories (Figure 3). The objective of the linking process is to translate the identified concepts into the most fitting ICF categories. Both researchers will have received prior, extensive linking training by the ICF Research Branch. Any discrepancies between the two reviewers will be resolved through discussion, or with the involvement/consultation of an independent third reviewer. Finally, a frequency analysis will be done on the linked categories. An ICF category that appears in a publication multiple times will be counted only once. The ICF categories identified in at least 5% of the publications will be included in the list of candidate categories.^33^

#### Preparatory study 2: Qualitative study (patient and/or caregiver perspective)

The qualitative study aims to explore experiences of adults living with multimorbidity (and their caregivers) in relation to functioning.^33^ At least one focus group will be conducted in each of the six WHO regions (Africa, Europe, the Americas, Eastern-Mediterranean, South-East Asia, and Western Pacific). Where health or logistical challenges prevent an individual from participating in a focus group, individual interviews will be conducted.^42^ These interviews and/or focus groups will be conducted in participants’ primary language, in person (e.g., at health centres or patients’ homes) and/or online, by local clinician co-investigators/collaborators (preferably rehabilitation professionals) recruited through existing research platforms, networks, and relationships with healthcare facilities in the participating countries in the six WHO regions.

In previous preparatory qualitative studies of ICF Core Sets,^43–46^ four to six focus group discussions were required to achieve data saturation.^47^ This study will include approximately six focus groups with the final number determined by saturation. In each participating country, focus groups and interviews will be conducted by two local clinician co-investigators/collaborators together (i.e., one as moderator and the other as an assistant). These professionals will be supported to recruit participants primarily at the hospitals/clinics where they work, during participants’ regular hospital visits. Eligible participants include individuals with multimorbidity and/or their caregivers aged at least 18 years who provide written informed consent. Inclusion of caregivers will help prevent exclusion of individuals with insufficient decisional capacity such as those with cognitive impairments. This is because caregivers can provide researchers with proxy consent and information asked for by researchers about such individuals.^38^

The focus groups will comprise eight participants each, following a maximum variation strategy by considering factors such as age, sex, ethnicity, level of education, severity, and clusters of multimorbidity.^48^ A case record form (CRF) will be developed to capture participants’ socio-demographic information (e.g., date of birth, sex, marital status, living situation, formal education, and work status) and their multimorbidity (e.g., diagnoses and current treatments). After taking consent from the participants, the focus group and interview sessions will be audio recorded.

Focus groups and interviews will be semi-structured using a set of open-ended questions that address each of the six ICF components (Table 1) – body functions, body structures, activities and participations, environmental factors, and personal factors. Where appropriate, the moderator will use illustrative examples or probing questions to clarify the six ICF components.^42^ At the end of each ICF component discussion, a summary of the main results will be provided to the participants to review, verify, and amend the findings, as part of enhancing rigor of the data.^49,50^ All audio-recordings of the focus groups and/or individual interviews will then be transcribed verbatim and, where a language other than English was used, translated into English. The interviews and group discussions will last for a maximum of approximately 60 and 120 minutes, respectively, to complete (including short breaks where necessary).^42^

**Table 1.** ICF-based interview guide for the qualitative study.

ICF components	Open-ended questions
Body functions	How do your multiple long-term conditions (multimorbidity) affect the way your body and mind work?
Body structures	In which parts of your body do your multiple long-term conditions (multimorbidity) cause problems?
Activities and participation	How do your multiple long-term conditions (multimorbidity) affect the things you can and cannot do in your everyday life?
Environmental factors – facilitators	What and/or who in the environment where you live/work/study is helpful and supportive in your everyday life with multiple long-term conditions (multimorbidity)?
Environmental factors – barriers	What and/or who in the environment where you live/work/study make your everyday life with multiple long-term conditions (multimorbidity) difficult?
Personal factors	When you think about yourself and the person you are, what helps you to handle your everyday life with multiple long-term conditions (multimorbidity)?

Transcripts will be imported into NVivo software (QSR International, Massachusetts, USA) and analysed using qualitative content analysis.^51,52^ Two researchers will review each transcript. They will independently extract statements related to functioning which they will divide into meaningful units. Such units will be subsequently abstracted into concepts pertinent to functioning. The two researchers will compare generated concepts, and disagreement will be resolved by a third reviewer (MS, ICF Research Branch Coordinator). Thereafter, the two researchers will independently link the identified meaningful concepts to ICF categories using established ICF linking rules.^40^ The reviewers will then compare their linking results, resolve any discrepancies through discussions or involve/consult an independent third team member, and develop a final list of categories. Following analysis of each transcript, the research team will determine whether further participants need to be recruited for additional focus group sessions.^50^ This is to ensure data saturation, whereby linking of meaningful concepts in two consecutive focus groups or individual interviews results in no additional ICF categories.^50^

#### Preparatory study 3: Expert survey (health professional perspective)

The expert survey will explore expert perspectives on the problems faced by individuals with multimorbidity. The specific aims are^1^ to identify function-related problems that they find pertinent to address among patients with multimorbidity and^2^ to quantify these problems using the ICF. We will primarily recruit experts in rehabilitation such as physiotherapists, occupational therapists, speech and language therapists, prosthetists and orthotists, physical and rehabilitation medicine physicians, and audiologists. Eligible participants are those with at least two years of experience in research and/or assessment, treatment and care of persons with multimorbidity within their field of practice and/or research, from all six WHO regions.

Experts will be recruited through the authors’ professional networks complemented by an internet search to identify contact information of multimorbidity experts, also leveraging connections with professional societies, international organizations, academic centres, hospitals, university departments, social media platforms, and editorial boards of peer-reviewed journals, in all six WHO regions. We will reach out to prospective participants (and groups who may be able to propose relevant experts) via email with information about the study and an invitation to participate in the survey (they will be given three weeks to respond to the invitation, with a weekly reminder to prompt them to respond). Snowball sampling will also be used to identify further experts by requesting participants to recommend potential participants as part of the survey. We will finally publish the study information on relevant websites and social media platforms to recruit and collect data from experts online, who may have otherwise not been reached via the methods above.

The survey will be administered via both the World Wide Web- and email. It will initially be available in English and, where necessary, translated into a language a participant uses if not English. Experts who agree to participate will be asked to include their socio-demographic information in section one of the survey, including their discipline, work experience, and WHO region (to confirm their eligibility and ensure that a global perspective from diverse fields is captured). Section two will ask them six open-ended questions related to functioning of individuals with multimorbidity across the six ICF components (Table 2). Participants will be asked to complete and submit their survey responses within one month, with weekly reminders to do sent and submission deadline extension given if requested.

**Table 2.** ICF-based interview guide for the expert survey.

ICF components	Open-ended questions
Body functions	From your experience working with individuals with multiple long-term conditions (multimorbidity), how do multiple long-term conditions(multimorbidity) affect the way their body and mind work?
Body structures	From your experience working with individuals with multiple long-term conditions (multimorbidity), which parts of their body do multiple long-term conditions (multimorbidity) give them problems?
Activities and participation	From your experience working with individuals with multiple long-term conditions (multimorbidity), how do multiple long-term conditions (multimorbidity) affect the things they can and cannot do in their everyday life?
Environmental factors – facilitators	From your experience working with individuals with multiple long-term conditions (multimorbidity), what and/or who in the environment is helpful and supportive in their everyday life with multiple long-term conditions (multimorbidity)?
Environmental factors – barriers	From your experience working with individuals with multiple long-term conditions (multimorbidity), what and/or who in the environment is difficult in their everyday life with multiple long-term conditions (multimorbidity)?
Personal factors	From your experience working with individuals with multiple long-term conditions (multimorbidity), what personal characteristics or circumstances support their daily functioning with multiple long-term conditions (multimorbidity)?

Similarly to the development of ICF Core Sets for deafblindness (by WW and colleagues),^50^ this study aims to recruit approximately 100 experts, with the final sample size determined by saturation. To prevent overrepresentation of one WHO region over another, the team will aim to have a similar number of experts per region.

Expert survey responses will then be analysed to identify the meaningful concepts contained in them. Thematic^53^ or content^51^ analysis will be used. Like in the qualitative study (preparatory study 2), meaningful concepts will be extracted through line-by-line coding and then linked to ICF categories, using established ICF linking rules.^40,54,55^ To assure the quality and consistency of results, both the identification of meaningful concepts and the linking to ICF categories will be conducted by two independent researchers. These researchers will compare their linking results; discussions between them or consultation/involvement of a third independent researcher will be used to resolve any disagreements. Finally, a frequency analysis will be done on the linked categories. A category will be counted only once for each expert, even if several answers are given that will be linked to the same ICF category.^33^ ICF categories reported by at least 5% of the experts will be included in the list of candidate categories provided to participants of the subsequent international consensus conference.^33^

#### Preparatory study 4: Empirical study (clinical perspective)

Lastly in this phase, an international, multi-centre, cross-sectional study, will be conducted in at least one country within each of the six WHO regions, to identify common problems experienced by persons with multimorbidity in a clinical setting (preferably rehabilitation centres/wards).^33^ The same local clinician co-investigators/collaborators of the qualitative study (preparatory study 2) will conduct semi-structured interviews with a sub-set of patients with multimorbidity and/or their caregivers from the participant pool of the qualitative study (preparatory study 2), in the participants’ primary language, and rate their functioning using an extended ICF Checklist 2.1a.^56^ Prior to data collection, the clinicians will be trained in the administration of the ICF Checklist 2.1a, and its version of how participants’ responses are coded, and how the qualifiers are administered according to ICF rules.

The extended ICF Checklist 2.1a includes a demographic questionnaire and a selection of 125 categories covering all ICF components that the WHO considered most relevant for documentation in clinical practice.^57^ The checklist is made up of four parts that outline the following ICF-related areas: (a) impairments in body functions and structures, (b) limitations in activities and restriction from participation, (c) barriers and facilitators in the environment, and (d) other contextual information such as personal factors.^56^ To ensure that Checklist 2.1a captures a wide spectrum of possible relevant areas of patient functioning specific to individuals with multimorbidity, it will be supplemented with the candidate ICF categories resulting from the systematic literature review (specifically, categories covered by at least one of the most commonly used outcome measures), qualitative study, and expert survey, if those candidate categories are not yet included in ICF Checklist 2.1a.^58^

The clinicians will recruit and collect data from participants (patients with multimorbidity and/or their caregivers) during their regular hospital/clinic visits/consultations/admission. Eligible participants will be those with multimorbidity and/or their caregivers, aged 18 years or older, and willing to provide informed consent. Again, participant recruitment will follow a maximum variation strategy as previously described. The estimated number of participants required is about 100, as in previous studies with this design,^58–60^ but the final sample size in this study will be determined by saturation and participant representation across the six WHO regions.

The extended ICF Checklist 2.1a requires the investigator to rate the extent of a problem in each of the ICF categories or the extent to which an environmental factor is a facilitator or a barrier using the ICF qualifiers. The ICF provides qualifiers from 0 to 4 for each category. Because the metric properties of this scale are not yet evaluated sufficiently, we will use a dichotomized qualifier instead. Each category of the components Body Functions, Body Structures and Activities and Participation will be graded with the qualifiers 0 for ‘no impairment/limitation’, 1 for ‘impairment/limitation’. The categories of the component Environmental Factors will be graded with +0 or −0 for “No facilitator/no barrier”, +1 for ‘facilitator’ and −1 for ‘barrier’. Additionally, the qualifier ‘8’ will be used if the available information was not sufficient and ‘9’ if the category will not be applicable.

Descriptive statistics will be employed to analyse the qualifiers of the ICF categories from the completed ICF checklists and frequency with which specific ICF categories are reported as a problem (in the case of activity limitations and participation restriction), and barrier and/or facilitator (in the case of environmental and personal factors). An ICF category considered a problem, barrier, and/or facilitator by at least 20% of the studied patients and/or their caregivers (the cut-off will depend on the data collected) will be included in the list of candidate categories.^33,58^

The four preparatory studies above will generate four lists of candidate categories (i.e., one list from each study). These candidate categories will be consolidated to create a preliminary comprehensive ICF Core Set for multimorbidity rehabilitation.^50^

### Phase 2

#### International ICF Core Set consensus conference

Following guidance of the WHO and the ICF Research Branch,^33^ we will hold an online international consensus conference to complete the development process for the ICF Core Set for multimorbidity rehabilitation. This will be an iterative decision‐making and consensus process involving 21–24 experts (experience from previous consensus conferences verifies that the optimal number of participants is within this range^33^), including healthcare professionals and researchers working with adults with multimorbidity (preferably those within the rehabilitation field), and patient representatives, representing equal distribution across professional disciplines, WHO regions, and sex. The pool of participants from the expert survey above will be used as a source to identify and invite potential participants to the consensus process. The online decision‐making and consensus process will be conducted via Microsoft Teams, over a four‐week period with one‐ to three‐hour sessions per week.

Prior to the beginning of the consensus process, participants will be introduced to the essentials of the ICF. Furthermore, the process of developing ICF Core Sets, the results of the preparatory studies, and the consensus and decision-making process of the conference (including the voting process), will be presented. Participants who can also complete the ICF e‐learning tool provided by the WHO (https://www.icf‐elearning.com/) will be encouraged to do so. To help prepare the participants to contribute meaningfully to the consensus, a list of candidate categories aggregated from the four preparatory studies will be provided to them in advance of the conference, along with the request to select (in advance of the conference) the categories they thought were essential to include in the Comprehensive ICF Core Set for multimorbidity rehabilitation.

The introduction will then be followed by a structured, iterative decision-making process, involving alternating work group and plenary sessions, in which the participants discuss and vote on the categories to be included in the Comprehensive ICF Core Set. This process will proceed as per the detailed guidance of the WHO and ICF Research Branch.^33^ In summary, participants will be provided with the consolidated list of ICF categories resulting from a synthesis of the evidence gathered by the four preparatory studies. The final ICF Core Set will be reached based on affirmation percentages. ICF categories that will have been affirmed by at least 75% of the participants will automatically be included in the Comprehensive ICF Core Set for multimorbidity rehabilitation. Categories affirmed by less than 40% of the participants will be automatically excluded from the Comprehensive ICF Core Set. Categories will be considered “ambiguous” if affirmed by 40-70% of the participants. Such categories will be further discussed by the participants, providing arguments for or against including them in the Comprehensive ICF Core Set.

The comprehensive version will be the basis for developing the Brief ICF Core Set. In line with the guidance of the WHO and ICF Research Branch.,^33^ the development process for the Brief ICF Core Set will involve requesting participants to rank the most essential ICF categories from the Comprehensive ICF Core Set in three consecutive ranking sessions. In each ranking session, participants will be provided with a link to a Mentimeter listing all categories of the Comprehensive ICF Core Set and will be asked to individually rank the ten most essential categories for each ICF component in order of their importance. Holding three ranking sessions allows the participants to share the reasoning for their ranking decision and gives them an opportunity to re-assess their decision. The result of the individual rankings will be statistically calculated, and a common ranking will arise. Following the final ranking session, the process of deciding a “cut-off” for each ICF component will begin, at which time each participant will be asked to decide how many ICF categories per ICF component would be important to include in the Brief ICF Core Set. The consensus on the cut-off will then be applied to the final ranking results, and the second milestone will be reached – the Brief ICF Core Set for multimorbidity rehabilitation.

### Phase 3

#### Validation of the ICF core set

Finally, we will validate the first versions of the Comprehensive and Brief ICF Core Set developed above before they are introduced into practice.^33^ “*To ensure that the core sets are internationally valid and limit and clarify the impact of cultural context, it is important to implement the validation process in an internationally representative manner, similar to the preparatory studies to establish the core sets*”.^61^ We will use a validation process like that used by Van Leeuwen et al.^62^ In summary, we will compare our ICF Core Sets to ICF categories generated from the ICF-linked data of relevant primary multimorbidity studies for which authors/researchers are willing to share data (e.g., MultiLink study in Malawi, low-income country^63^; COSMOS study in 10 LMICs across South Asia, Latin America, and Western Africa^30^; PERFORM trials in the United Kingdom and Australia (HICs)^64^; etc).

Specific research questions for this validation study are:

• To what extent are our ICF Core Set for multimorbidity rehabilitation represented in the data sets of the other studies?
• Are there any extra ICF categories expressed in the data sets of the other studies that are currently not part of our ICF Core Set for multimorbidity rehabilitation, or are there extra constructs expressed in the data sets of the other studies that are not part of the ICF? If so, what are these constructs?

We will extract relevant content from the data sets of the other studies and link it to the most precisely corresponding ICF category using the WHO ICF linking rules.^40,54,55^ We will calculate descriptive statistics (frequencies, means, and SDs) for participants’ sociodemographic and condition-related characteristics. The extent to which the ICF Core Set for multimorbidity rehabilitation will be represented in the data sets of the other studies will be determined by assessing the overlap between our ICF Core Sets and the list of unique ICF categories extracted from the data sets of the other studies. Overlap will be expressed as percentage of ICF Core Set categories represented. We will also determine the extent to which there is no overlap. This will be the proportion of the ICF Core Set categories not represented in the data sets of the other studies. No overlap will also cover the extra (non) ICF categories that will be expressed in the data sets of the three studies but are not part of the ICF Core Set for multimorbidity rehabilitation. We will differentiate between non-ICF Core Set categories and constructs currently not part of the ICF.

A substantial overlap (what is “substantial” will be decided by the study team) between our ICF Core Sets and the data sets of the other studies will support content validity of our ICF Core Sets. On the other hand, no overlap will uncover areas that will not have been addressed in either the ICF Core Set or the data sets of the other studies. The results may suggest that either the data sets of the other studies or our ICF Core Sets may not cover all aspects that are relevant to patients with multimorbidity. The identification of extra constructs from the data sets of the other studies may suggest that our ICF Core Set do not include all areas of functioning that are relevant to multimorbidity rehabilitation. Controversially, it could also suggest that the researchers of the other studies picked constructs that are not relevant for multimorbidity rehabilitation, which would not be surprising for some of the other studies which were not specifically conducted in the context of rehabilitation (e.g., COSMOS) Nevertheless, this comparative data analysis will potentially strengthen a recommendation to implement our ICF Core Sets and/or inform their revision before implementing them in practice.

## Conclusion

An ICF Core Set created for multimorbidity rehabilitation will^1^ benefit patients with multimorbidity who are often excluded from clinical trials of single-disease rehabilitation programs^65,66,^^2^; ensure precise and comprehensive assessment and documentation of functioning aspects relevant to this patient population^3^; help rehabilitation providers and their patients and/or caregivers when setting rehabilitation goals and planning rehabilitative interventions to achieve those goals^4^; help researchers in synthesis of evidence for multimorbidity rehabilitation and facilitate the comparability of data across studies and countries: and^5^ provide the scientific basis from which assessment tools can be derived for use in clinical and research settings, as well as in health care administration.^42^

## Data Availability

The datasets generated and/or analysed during this research project will be Available from: the corresponding author upon reasonable request. *

## Trial status

The project has been registered with the Core Outcome Measures in Effectiveness Trials (COMET) at https://www.comet-initiative.org/Studies/Details/3266. The extraction of outcome measures from the studies included in Bricca et al. (2) and Barker et al.(23) systematic reviews has been completed by JM, and we will next be linking their underlying concepts to the ICF categories. For the validation phase, discussions with the COSMOS team (University of York), MultiLink team (Malawi-Liverpool-Wellcome Programme/Liverpool School of Tropical Medicine), and PERFORM (University of Leicester) regarding data sharing agreements have been initiated.

## Dissemination plan

We will publish manuscripts reporting findings of individual studies from each phase in open-access peer-reviewed journals (we anticipate at least six publications). We will present our findings at international conferences including at the WHO Family of International Classifications (WHO-FIC) Network Annual Meeting and at the Annual Scientific Meeting of the Global Alliance for Chronic Diseases (GACD). We will publish our ICF Core Set on the ICF Research Branch webpage (https://www.icf-research-branch.org/). We will create an independent website specifically for our project.
